# Aggrecan-related bone disorders; a novel heterozygous *ACAN* variant associated with spondyloepimetaphyseal dysplasia expanding the phenotypic spectrum and review of literature

**DOI:** 10.1016/j.jgeb.2023.100341

**Published:** 2024-01-30

**Authors:** Hoda A. Ahmed, R. Elhossini, M. Aglan, Khalda Amr

**Affiliations:** a Medical Molecular Genetics Department, Human Genetics and Genome Research Institute, National Research Centre, Egypt; b Department of Clinical Genetics, Human Genetics and Genome Research Institute, National Research Centre, Egypt

**Keywords:** Skeletal dysplasias, ACAN, Heterozygous

## Abstract

**Background:**

Spondyloepimetaphyseal dysplasias (SEMD) are a large group of skeletal disorders represented by abnormalities of vertebrae in addition to epiphyseal and metaphyseal areas of bones. Several genes have been identified underlying different forms. *ACAN* gene mutations were found to cause Aggrecan-related bone disorders (spondyloepimetaphyseal dysplasias,spondyloepiphyseal dysplasias, familial osteochondritis dissecans and short stature syndromes). This study aims to find the disease causing variant in Egyptian patient with SEMD using whole exome sequencing.

**Methods:**

Whole-exome sequencing was performed for an Egyptian male patient who presented with short stature, clinical and radiological features suggestive of unclassified SEMD.

**Results:**

The study identified a novel de novo heterozygous *ACAN* gene variant (c.7378G>A; p.Gly2460Arg) in G3 domain. Mutations in *ACAN* gene have been more commonly associated with short stature than SEMD. The phenotype of our patient was intermediate in severity between spondyloepiphyseal dysplasia presentation; Kimberley type(SEDK) and Spondyloepimetaphyseal dysplasias Aggrecan (SEMDAG)

**Conclusions:**

Whole exome sequencing revealed a novel de novo *ACAN* gene variant in patient with SEDK. The clinical and skeletal phenotype of our patient was much severe than those reported originally and showed more metaphyseal involvement. To the best of our knowledge, two previous studies reported a heterozygous variant in *ACAN* with spondyloepiphyseal dysplasia presentation; Kimberley type.

## 1 Background

Skeletal dysplasis (SD) are consisting of over 400 heterogenous genetic disorders caused by congenital defects affecting the articular cartilage and bone development.^1^ Mutations affecting of the extracellular matrix proteins,cation channels, post-translational processing or chromatin regulation were the most frequent genetic causes of different forms of SEMDs. Spondyloepiphyseal dysplasias (SED) are characterized by chondrodysplasias with short-trunk disproportionate dwarfism.^2^, ^3^ Variants in the type II collagen (*COL2A1*) and the sedlin (*SEDL*genes result in two main subtypes of SEDs; autosomal dominant SED congenita (OMIM 183900) and X linked recessive SED tarda (OMIM 313400) respectively. Later on, many subtypes were added by the latest International Nosology and Classification of Constitutional Disorder of Bone.^4^ SED type Kimberley (SEDK; OMIM 608361), caused by variant in *ACAN* gene, is a rare mild autosomal dominant type of SED that was first recognized in a white descent South African family.^5^

Aggrecan is the primary sulfated proteoglycan in the cartilage and is also expressed in the brain, aorta, and tendons. It has negative charge ions which responsible for attraction of water,permitting hydration of cartilage to resist the high mechanical load present in the skeletal joint.^2^ In addition, it is fundamental in particular chondrocyte ossification and bone morphogenesis. Aggrecan abnormalities result in defect in chondrocyte differentiation and endochondral ossification at the growth plate leading to a large heterogenous group of skeletal diseases.^6^ Aggrecan protein is encoded by *ACAN* gene which is located on chromosome15 and has 19 exons. The core protein consists of centrally located glycosaminoglycan attachment domain (GAG), three globular domains (G1,G2, and G3) and interglobular domain (IGD). G3 domain is considered the most important domain as it consists of two epidermal growth factor-like repeats, followed by a C-type lectin domain (CLD) and a complement regulatory protein repeat.^7^, ^8^ Variants in *ACAN* gene have been identified in humans and other species and is associated with broad spectrum of phenotypes ranging from short stature to severe skeletal dysplasias. In mice, cattle and chick, complete lack of aggrecan has been reported to cause severe chondrodysplasia.^9^, ^10^

Aggrecan-related bone disorders are a heterogeneous group of diseases caused by variants in *ACAN* gene, including spondyloepimetaphyseal dysplasia, aggrecan type (OMIM 612813), macrocephally with multiple epiphyseal dysplasia and distinctive facies (OMIM 607131), spondyloepiphyseal dysplasia, Kimberley type (SEDK; OMIM 608361), familial osteochondritis dissecans (OMIM 165800) and idiopathic short stature. In many studies, multiple *ACAN* gene variants have been identified to cause isolated short stature.^6^, ^8^ The first identified family with SEDK followed an autosomal dominant inheritance caused by (c.3986dupC; Gly1330Trpfs*221) in *ACAN* gene.^11^ Our study aims to identify the genetic etiology in a proband with SEMD using whole exome sequencing.

## 2 Methods

The study investigated an Egyptian male patient, who presented to the Limb Malformations and Skeletal Dysplasia Clinic (LMSDC), National Research Centre at the age of 8.5 years with short stature and skeletal defects. Clinical examination, anthropometric measurements and radiological investigations were performed. Ethical procedures were approved by the Medical Research Ethics Committee of the National Research Centre, Egypt according to the Declaration of Helsinki. The parents signed written informed consents to be included in our study.

## 3 Molecular analyses

### 3.1 DNA Extraction and whole exome sequencing

Extraction of DNA from peripheral blood samples of the proband and his parents was done using QIAamp DNA Mini Kit (Qiagen, Germany). the purity and concentration of DNA samples were assessed using NanoDrop ND-100. The coding and flanking intronic regions were enriched using in solution hybridization technology and were sequenced using the Illumina HiSeq/NovaSeq system (Illumina, CA, USA) according to the manufacturer’s protocol.^12^, ^13^ The detected variants were annotated using ANNOVAR then filtered according to depth of coverage and minor allele frequencies (<1%) in many databases (1000 Genomes Project**,** and the Genome Aggregation Database).^14^, ^15^, ^16^, ^17^, ^18^ We focused on variants in SMED genes then the filtration of causative variant was performed based on mode of inheritance, gene function and predicted damaging effects using in silico prediction tools.

### 3.2 Variant segregation

Sanger sequencing was performed to verify that pathogenic variant segregated through parents. Primer3 tool was used to design forward and reverse primers targeting *ACAN* exon which contains the detected variant.^19^ PCR was carried out as previously described.^20^ Sequencing was done on ABI Prism 3500 Genetic Analyzer (Applied Biosystems, USA) according to manufacturer’s recommendation using Big Dye Termination kit (Applied Biosystems, USA).

### 3.3 In silico prediction of protein changes created by *ACAN* variant

Different In silico tools including PREMPS **(**https://lilab.jysw.suda.edu.cn/research/PremPS), SAAMBE3D (https://compbio.clemson.edu/saambe), DynaMut2 (https://biosig.unimelb.edu.au/dynamut) and Missense3D (https://missense3d.bc.ic.ac.uk/missense3d) were used to predict the effect of the detected variant on protein structure (Table 1). No Protein Data Bank (PDB) crystal structure of ACAN was found so we recovered a predicted protein structure model covering the whole ACAN amino acid chain created by AlphaFold from the Uniprot database (ID: P16112) (Fig. 2). PremPS predicts the effect of missense mutations on protein stability by calculation the difference in the unfolding free energy (ΔΔG) in each mutated protein. Missense3D identifies changes in conformational features such as cavity volume, buried versus exposed states of target residue and changes in charge and hydrophobicity. SAAMBE-3D predicts the effects of single amino acid mutation on protein–protein interactions (PPIs) through prediction of binding free energy change caused by a variation. Dynamut assesses alteration of protein stability and flexibility.

**Table 1:** In silico variant effect prediction tools and ACMG classification of *ACAN* variant.

**Gene**	**Mutation**	**Polyphen** **Score**	**PhD-SNP** **score**	**Mutation Taster**	**PROVEAN** **Score**	**Sift** **Score**	**CADD** **Score**	**MUpro** **Score**	**MutPred** **Score**	M- **CAP** **Score**	**EIGEN** **Score**	**ACMG Classification**
***ACAN*** **(NM_013227.3)**	c.7378G > A(p.Gly2460Arg)	Probablydamaging(1.000)	Disease(RI 8 )	Diseasecausing	Damaging( 7.11)	Deleterious(0.02)	(23.1)	detal delta G = −0.6626496(decrease stability)	Pathogenic(0.6)	Damaging0.1316	Pathogenic0.4105	Uncertain Significance(PM2 PP3)

## 4 Results

### 4.1 Clinical features

**Fig. 1:**
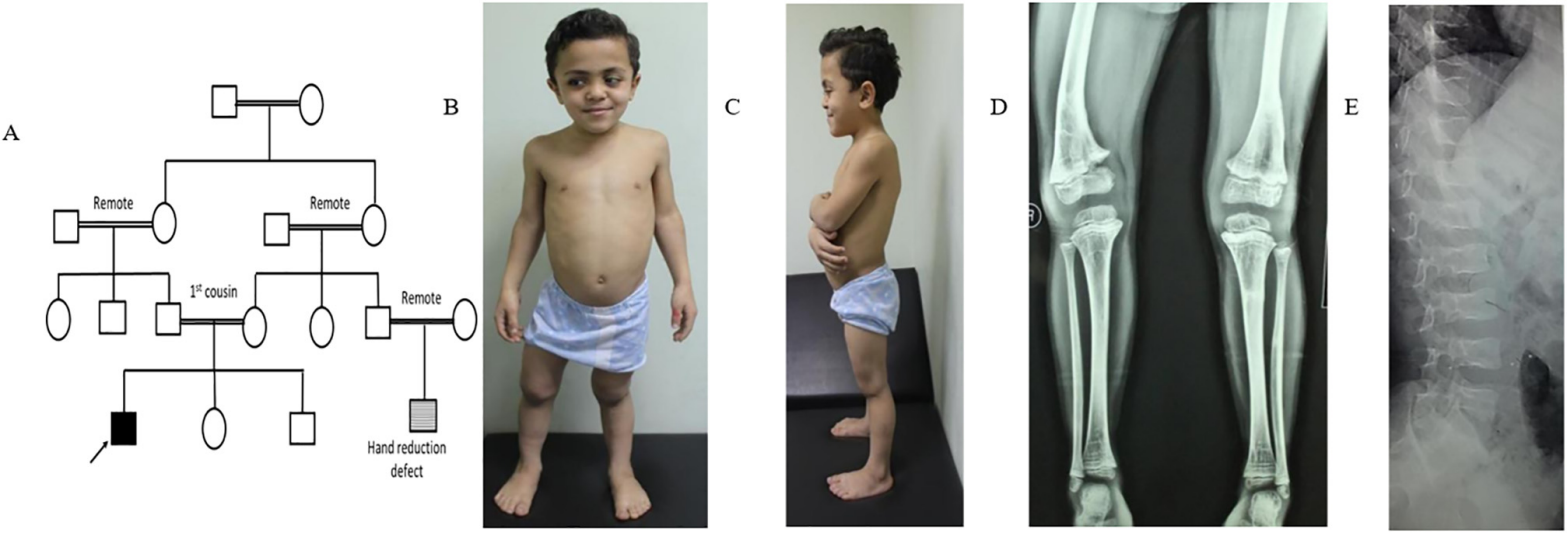
**A**; Four generation family pedigree. **B** and **C**; Patient at the age of 8.5 years, frontal view and lateral view showing relative macrocephaly, high forehead, hypertelorism, flat face, protuberant abdomen, lordosis, tilted pelvis, genu recurvatum and genu valgus mainly at left knee. **D**; X-ray lower limbs showing mild femoral bowing, abnormal metaphyseal trabeculations, wide irregular metaphysis and flat epiphysis. **E**; X-ray vertebrae (lateral view) showing platyspondyly.

The proband presented to the LMSDC at the age of 8.5 years with short stature, skeletal deformities and painful walking that started at early childhood. The patient descends from positive consanguineous family with family history of a cousin who had left hand reduction defect. Birth weight was 3Kgs (−0.9SD). He has average mentality and normal achievement of motor development. Clinical examination revealed high forehead, flat face, depressed nasal bridge, short neck, lordosis,mild scoliosis, lax joints, swan neck deformities at interphalangeal (IP) joints, and genu valgus associated with genu recurvatum. The external genitalia were normal and neurological examination was free. Anthropometric measurements of the patient revealed that his weight was 18 kgs (−2.76SD), his head circumference was 51 cm (−1.03 SD), arm span was 102.8, he was short (101 cm) (−5.02 SD), and sitting height was 56 cm. He had an increased upper segment to lower segment ratio (1.25); sitting height to height ratio was 0.55, indicating mild lower limb affection. Echocardiography and electroencephalogram were normal. Skeletal survey revealed platyspondyly, flat acetabulum, wide irregular metaphysis, abnormal metaphyseal trabeculations and epiphyseal flattening in addition to decreased bone density and delayed bone age (bone age was equal to 5 years and 6 months at presentation) **(**Fig. 1**)**. History taking revealed that the patient discontinued growth hormone after1 year of treatment as no response was detected moreover he was operated for genu valga twice with recurrence. Comparison between heterozygous ACAN-positive patients (SEDK) and homozygous ACAN-positive patients (SEMD Aggrecan) with our patient are summarized in Table 2.

**Fig. 2:**
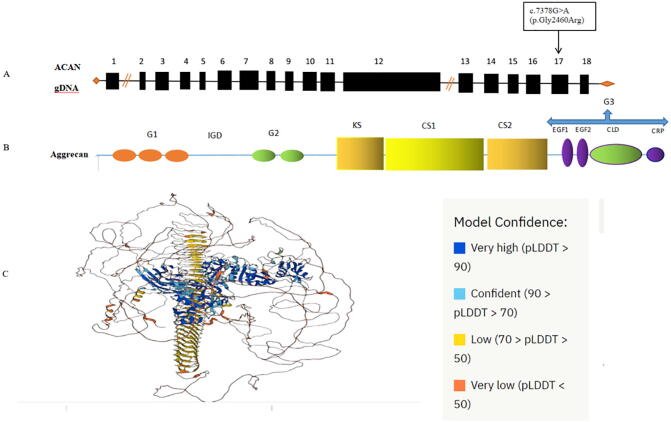
*ACAN* gene structure and location of detected variant in exon17. **B.** Structure of ACAN aggrecan protein. **C.** Predicted 3D structure of the ACAN protein.

**Table 2:** A comparison between heterozygous *ACAN-*positive patients (SEDK) and homozygous *ACAN-*positive patients (SEMD Aggrecan) with our patient.

**Characteristic**	**Reported heterozygous** ***ACAN-*positive patients (SEDK)** *Anderson et al. 1990* and *Sentchordi-Montané et al. (2018)*	**Reported homozygous *ACAN-*positive patients (SEMDAG)** *Tompson et al. (2009)* *and* *Fukuhara et al. (2019)*	**Our patient**
**Mentality**	Average	Average	Average
**Motor development**	Normal	Normal	Normal
**Type of short stature**	Proportionate short stature with a stocky habitus	Severe short limbs short stature	Mild short limbs short stature
**Degree of short stature**	Mild to moderate	Extreme	Severe (-5.02 SD)
**Facies**	Not dysmorphic	Severe midface hypoplasia in one family	High forehead, flat face and depressed nasal bridge
**Beck deformities**	Lordosis	Lordosis and kyphosis	Lordosis and scoliosis
**Lower limbs deformities**	Premature arthropathy	Premature arthropathy	Genu valgus and genu recurvatum
**Brachydactyly**	Short metacarpals in one family	Severe	No
**Joints laxity**	ND	Present	Present
**Platyspondyly**	Present	Present	Present
**Epiphyseal dysplasia**	Flat irregular epiphysis	Flat irregular epiphysis	Flat epiphysis
**Metaphyseal dysplasia**	ND	Wide metaphysis	Wide irregular metaphysis with trabeculations
**Bone age**	Delayed in one patient	Advanced	Delayed

### 4.2 Molecular data and bioinformatics results

**Fig. 3:**
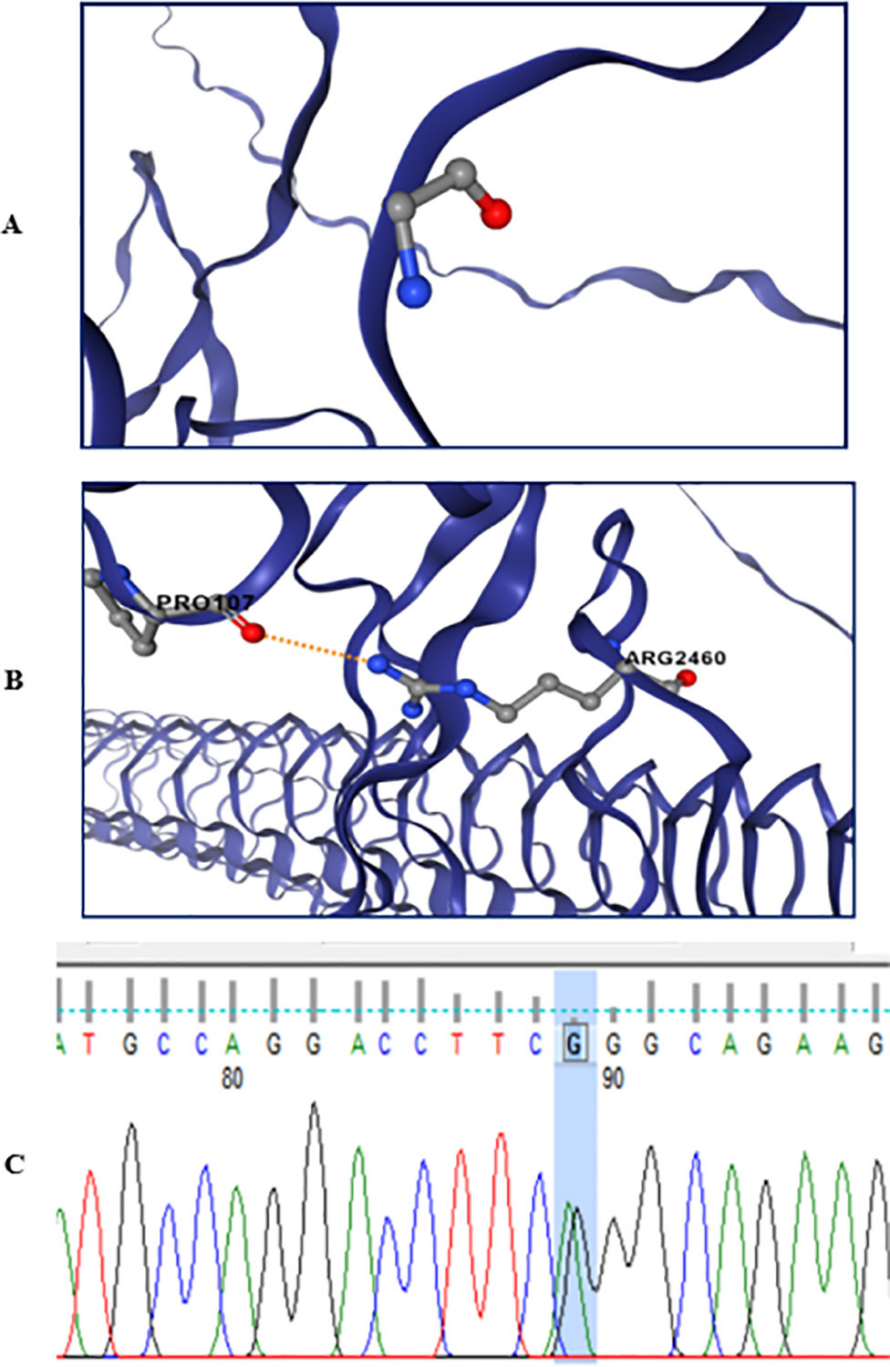
Prediction of alteration of protein structure by *ACAN* variant c.7378G > A (p.Gly2460Arg) using The PremPS tool. (**C)** Sanger sequencing chromatograms of exon 17 of *ACAN* gene in our patient.

Exome analyses of the studied patient revealed a novel de novo heterozygous missense *ACAN* variant (c.7378G>A; p.Gly2460Arg) in exon 17 in C-type lectin domain (CLD) of G3 domain. (Fig. 2) This variant was not previously reported in any of the databases. Sanger sequencing was performed to confirm the variant (Fig. 3). Variant segregation of the parents showed the presence of wild-type alleles of the gene. The p.Gly2460Arg is classified as uncertain significance variant based on ACMG guideline. The variant was predicted to be deleterious by different in silico algorithms (Table 1). CADD scores were greater than 20, which means that this variant is among the top 1 % of deleterious variants of the human genome. The identified *ACAN* variant is located in highly conserved regions in functional protein G3 domain. This was also evident in their positive GERP scores, which refers to constrained genomic regions across different species. The glycine residue at codon 2460 of ACAN is conserved in all mammalian species.

The PremPS tool predicted that the missense (c.7378G>A; p.Gly2460Arg) variant was destabilizing, with ΔΔG values of 0.52 kcal/mol **(**Fig. 3**)**. The Arg2460 residue introduces destabilizing conformational changes because there is a moderate physicochemical difference between glycine (Nonpolar hydrophobic) and arginine (basic hydrophilic). Missense 3D software predicted Gly2460Arg mutation to produce contraction of the cavity volume by 87.7 Å^3^ and trigers disallowed phi/psi alert. The phi/psi angles are in the favored region for wild-type residue but outlier region for mutant residue with consequential damaging conformational changes. SAAMBE displayed that the G2460R destabilize the protein with ΔΔG 0.68 kcal/mol. DynaMut 2 disclosed that G2460R mutation led to decrease of molecule flexibility with ΔSVib ENCoM: −0.29 kcal.mol^−1^.K^−1^.

## 5 Discussion

**Fig. 4:**
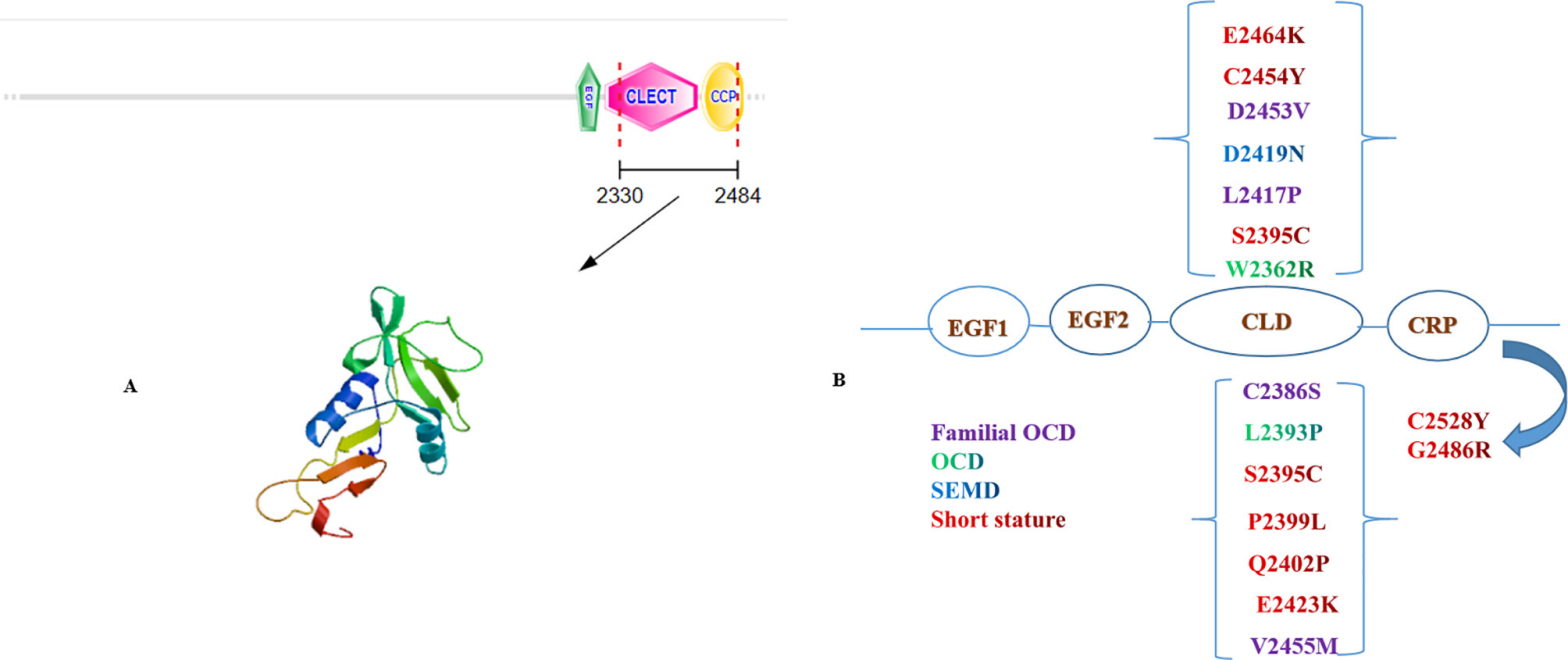
**A:** Predicted 3D structure of the aggrecan G3 domain **B**: G3 domain missense mutations. Familial OCD Variants are shown in purple, while SEMD and SEDK variants in blue, OCD are shown in green and variants linked to short stature are shown in red. (For interpretation of the references to colour in this figure legend, the reader is referred to the web version of this article.)

Aggrecan is a major proteoglycan in articular cartilage having numerous sulfate and keratan chains encoded by the *ACAN* gene. Aggrecan is fundamental in articular cartilage, chondrocyte and bone morphogenesis. Different phenotypes were caused by different types of variants in ACAN gene known as Aggrecan- related bone disorders.^6^ In human, heterozygous *ACAN* variants have been identified in SEDK, and autosomal dominant familial osteochondritis dissecans, whereas homozygous mutations result in SEMDAG and macrocephaly with multiple epiphyseal dysplasia and distinctive facies.^9^, ^10^, ^21^ All missense mutations in G3 domain result in broad spectrum of aggrecanopathies (Fig. 4), this clinical heterogeneity resulted from presence of different disease mechanisms or modifier genes. Modifier genes could affect aggrecan expression levels or post- translational processing.^9^ Two different disease mechanisms were reported to be involved in the aggrecan disease spectrum, first; premature termination codons resulting in truncated proteins. Second mechanism was alteration of structure and function of the cartilage through dominant-negative missense mutations.^9^ Herein, we detected a family with SEMD, caused by a novel de novo heterozygous *ACAN* variant (c.7378G>A; p.Gly2460Arg) in exon 17 in G3 domain. More than 90 variants have been identified in *ACAN* gene, most of them were associated with idiopathic short stature (ISS).^2^ Nineteen % of *ACAN* variants were clustered in exon 12; 87.9 % of these variants were inherited whereas the remaining were de novo.^3^

Based on previous studies on animal models, G3 is involved in regulation of attachment of GAG chains, secretion of aggrecan and it was fundamental for proper protein function.^9^, ^10^ **Kiani et al. (2002)**, disscused the role of G3 domain in GAG modification and product secretion and disclosed that cysteine residues in the CRD and CBP motif influence product secretion. To date, three affected families have been reported to have heritable form of SEDK caused by *ACAN* mutation, and in the current study, we reported the fourth family with one affected patient however, he had obvious metaphyseal involvement. The clinical and skeletal phenotype in our case is more severe than the three originally reported cases with SEDK. SEDK-heterozygous ACAN-positive individuals were the South African family of U.K (c.3986dupC; Gly1330Trpfs*221) and the Spanish, Portuguese families (c.-7-2A>C; c.1598C>T p. (Thr533Ile). The affected individual in the South African family had proportionate short stature (5th percentile for age), stocky build, and early onset osteoarthritis. Radiographic features were flattened vertebral bodies and femoral epiphyses, with sclerosis.^22^

Later, **Sentchordi-Montané et al. (2018)** reported a large study of heterozygous *ACAN*-positive individuals including Spanish and Portuguese families with SEDK. The clinical and radiological manifestations were variable, height ranged between −0.76 SD and −4.2 SD. Short neck, stocky habitus, obesity, limited elbow extension, waddling gait, platyspondyly and curved radius were reported. One patient had brachydactyly and irregular femoral epiphysis.

SEMD AG-homozygous ACAN-positive individuals were described by **Tompson et al. (2009)** in three affected sibs in a Mexican family **(25)**. The sibs, aged 16, 19, and 24 years, had short stature (66, 63 and 71 cm), macrocephaly, midface hypoplasia with no nasal cartilage, prognathism and low-set posteriorly rotated ears. They also had rhizomelia, mesomelia with no bowing of any segment and severe brachydactyly with telescoping interphalangeal joints. Radiographic features included long bones with generalized irregularities of the epiphyses with widened metaphyses, platyspondyly and multiple cervical-vertebral clefts. The carrier parents and half-sister were short.^23^ Later on, **Fukuhara et al. (2019)** reported the 2nd family of SEMD Aggrecan in a 45- year-old man. He was born to non-consanguineous parents and he had short stature (118.3 cm) (-9.1SD), relative macrocephaly,moderate platyspondyly with cervical lordosis and thoracolumbar kyphosis. No prognathism, low-set ears or midface hypoplasia were noted. Rectangle shape of vertebral bodies with shallow acetabulae were observed in this patient. Large joints had degenerative joint disease especially the hips. Long bones were short and relatively broad with metaphyseal flaring wth generalized brachydactyly.

**Sentchordi-Montané et al. (2018)** reported 2 patients with heterozygous *ACAN* variants with short stature and mild bone deformities, who had poor response to growth hormone therapy as in our patient. However, **Gkourogianni et al. (2017)** reported positive height response to GH in five heterozygous *ACAN* variants with short stature. The clinical manifestations of the Egyptian patient in our study were a milder phenotype of SEMDAG and more severe phenotype than SEDK.

## 6 Conclusions

In our study we reported a novel de novo variant in *ACAN* gene causing SEDK in an Egyptian patient. To date, this is the fourth family reported with SEDK and presenting with more skeletal manifestations than originally reported and metaphyseal involvement.

## Ethics approval and consent to participate

The study was approved by the Medical Research Ethics Committee of the National Research Centre (NRC), Cairo, Egypt. Informed consent was obtained from all individual participants included in the current study.

## Funding

The work was supported by Science and Technology development Fund (STDF).

## Author contribution statement

Elhossini R, Aglan M examined the patient and took the family and medical histories. They were assigned for documentation of the family details and the clinical photos. Hoda A. Ahmed, Khalda Amr collected the DNA samples, Sanger sequencing and data analysis. The manuscript was written and reviewed by all authors.

## Availability of data and material

All data generated or analysed during this study are included in this published article [and its supplementary information files].

## Declaration of competing interest

The authors declare that they have no known competing financial interests or personal relationships that could have appeared to influence the work reported in this paper.
